# Comment on Lansing/de Vet’s paper

**DOI:** 10.1007/s10745-012-9497-0

**Published:** 2012-06-04

**Authors:** Brigitta Hauser-Schäublin

**Affiliations:** Institute for Cultural and Social Anthropology, University of Göttingen, Theaterplatz 15, 37073 Göttingen, Germany

Lansing/de Vet’s article tries to substantiate the conclusion Lansing has presented in all his publications since “Priests and Programmers”, namely that the irrigation management was exclusively performed by water temples and their commoner priests. These water temples accomplished a synchronization of irrigation agriculture that was a means to achieve the sharing of water (up-stream/down-stream) and pest control. Furthermore, he suggested that subak (irrigation associations) have been democratic organizations run exclusively by farmers; no lords or rulers were involved in the management of irrigation agriculture. Lansing’s theoretical approach constitutes a grid which has been applied to the colonial literature in a deductive way and to data collection during fieldwork. The submitted article documents this perspective too.

Three issues have come under criticism from different scholars: A) The allegedly democratic organization of irrigation agriculture, the non-involvement of lords and the commoner-based water temples (Schulte Nordholt, myself). B) The pest control as a goal of the water temple management and the synchronization of water use (Vayda, Falvo). C) The way Lansing has arbitrarily made use of sources (theory-driven reading of sources; “confirmation bias”) so that they are made to fit his purpose (myself, Schulte Nordholt, Vayda).

In the submitted article, the authors react to critiques only in a restricted way; mainly to two articles published in Human Ecology 39,1 2011 and to a chapter by Vayda (2009). They react to A) and B), but not to C). The arguments put forward hardly convey new insights. The figures and lists of subak provided barely contribute to the clarification of the issues debated. The abstract seems to belong to another article (with the exception of the last two sentences).

Not only shorter quotations, but also longer ones, are without references, for example, the quotations from Vayda and Schulte Nordholt.

In the article, the authors attempt to defend the core statements Lansing has obstinately repeated over the past 20 years and to knock out all counter arguments. It is certainly due to this endeavor that contradictions in the paper have escaped the authors. On the one hand, the authors disclaim the historicity of (written) chronicles (ftn. 11). They do so in order to devalue my interpretation/conclusion. On the other hand, the authors use poetry as historical texts: “Kidungs are a metrical genre of Balinese literature, rich with metaphors and allusions, interpreting them is regarded as a specialized skill”. They use this text in order to substantiate once again one of their claims, namely the non-involvement of lords in the management of irrigation and the construction of dams, and to refute Schulte Nordholt’s argument about the royal involvement in irrigation management. The authors are rendering part of this literary text (a page long) and then simply pick out a couple of words they need for their argument; they do not specify what their methodological qualification is of what they call “specialized skill” needed for the kidung’s interpretation. They also do not refer back to Schulte Nordholt, who has dealt extensively with this kidung and its interpretation in his 1996 book.

In their effort to prove that Lansing’s conclusions are irrefutable, the authors extensively quote passages of a random selection of reports of earlier centuries on Bali. They do so without discussing the criteria for their selection and without evaluating these sources and the information they contain. One of the examples is a quotation from Raffles (1817), that is, before detailed research in Bali began. Raffles was a political economist following in the footsteps of Adam Smith. Raffles had, as Vickers notes, a “disdain for the despotic ‘feudal’ kings of the Indies which partly derived from earlier European images of Oriental despotism” (1989:21–22); no wonder that Raffles described the soil as “private property of the subject” - the soil of the “farmers” in Lansing’s terminology and theory.

In the submitted article, Lansing/de Vet also twist sources and references (a practice Lansing had already performed earlier, see Hauser-Schäublin 2003) in order to diminish the significance and validity of statements other scholars have made and to support their own assertions. Lansing/de Vet insinuate, for example, that Schulte Nordholt was unaware of the fact that the term subak had already appeared in inscriptions of the 11th century. This is simply ridiculous. None of the critics, and certainly not Schulte Nordholt, has ever doubted the old age of subak as a term, but the critics have challenged Lansing’s assumption that subak has always meant the same, and that subak preserved an unchanged organizational form for 900 years, as Lansing suggests.

Furthermore, the authors suggest in a similar, rather unfair, way that Schulte Nordholt’s arguments about the dynastic involvement in irrigation management is based on only four written sources, a kidung and “interviews recorded during his own fieldwork”. What Lansing/de Vet do not say is that Schulte Nordholt’s 2011 article is based, as this author I clearly spelled out (2011:21), on his 1996 book in which he dealt extensively with the questions of the involvement of lords in irrigation management, substantiated with comprehensive references from colonial literature, the results of his own extended archival research and his long periods of fieldwork.

Another example of how the authors twist the arguments critics have put forward concerns Vayda’s assessment. Vayda’s critique is primarily about methodology and theory. Vayda criticizes that Lansing excluded alternative possibilities of explanations from the beginning. He describes Lansing’s approach as “confirmation bias” (2009:46), that is “easy answers by indulging in consequence explanations”. Vayda then gives examples of what he means by Lansing’s “confirmation bias”, and discusses other possible causes than those given by Lansing for the pest explosion in the context of the Green Revolution. Lansing/de Vet misquote Vayda when they write: “But Vayda’s question is, were pests a problem before the Green Revolution?”. They go on to quote colonial officers who noted rice pests, such as mice and rats, invading the fields. Lansing/de Vet then describe pest control in the form of the ritual cremation of rats which, as they maintain, “sometimes accompany widespread synchronous fallows”. However, Vayda did not ask the question as rendered by Lansing/de Vet. Instead, Vayda simply stated that “the two main insect pests [green leaf hopper and brown plant hoppers] of Balinese rice fields after the Green Revolution in the 1960s, were much less a threat to the fields in earlier times” (2009:40). Thus, Lansing/de Vet try to evade the criticism by indulging in an answer to nothing raised by Vayda at all.

The same - twisting critiques so that they are easy to invalidate - applies to the way Lansing/de Vet quote my work. They pick up the term “royal temple” and insinuate that I referred to a single king as owner of the major water temple. The authors then go on to disprove this alleged suggestion by quoting a colonial text that contradicts it. Similar to Schulte Nordholt’s case, they do not refer to the various publications in which I described how different dynasties and lords were represented in the temple and were competing for supremacy (for example, 2003, 2005, 2008, 2011). They again suggest that my arguments were “entirely based on a literal interpretation of the myths [!] recorded in lontar manuscripts” (ftn. 11), though my results were based also on a multi-methods approach, as already explained in my 2003 article (and subsequent publications).

Another strategy applied by the authors to invalidate criticism is to truncate statements, for example, when they write that I proposed “that the temple of Batur was created by Javanese nobles from Majapahit”, while the original runs “the Batur Temple is the result of the colonizing efforts of immigrant Javanese nobles from Majaphit” (2011:46), with references to a more detailed analysis of the circumstances in an earlier publication.
